# Central Adiposity and Subclinical Cardiovascular Disease in Police Officers

**DOI:** 10.1155/2013/895687

**Published:** 2013-01-15

**Authors:** Penelope Baughman, Desta Fekedulegn, Michael E. Andrew, Parveen Nedra Joseph, Joan M. Dorn, John M. Violanti, Cecil M. Burchfiel

**Affiliations:** ^1^Epidemic Intelligence Service, Centers for Disease Control and Prevention, Atlanta, GA 30333, USA; ^2^Biostatistics and Epidemiology Branch, Health Effects Laboratory Division, National Institute for Occupational Safety and Health, Centers for Disease Control and Prevention, Morgantown, WV 26505, USA; ^3^The University of Chicago Comprehensive Cancer Center, The University of Chicago, Chicago, IL 60637, USA; ^4^Department of Social and Preventive Medicine, School of Public Health and Health Professions, The State University of New York at Buffalo, Buffalo, NY 14214, USA

## Abstract

Given the associations between obesity and cardiovascular disease (CVD), we evaluated a related but less well-established association between waist circumference and brachial artery reactivity (BAR), a functional measurement of subclinical CVD, where lower levels indicate dysfunction. Regression models examined trends in mean BAR across waist circumference tertiles in police officers, a high-stress occupational group with increased risk for CVD. Mean BAR decreased across increasing waist tertiles among men, but not women, and this association was stronger among officers who consumed more alcohol. Larger waist circumference may be associated with lower BAR, providing an opportunity for intervention prior to disease development.

## 1. Objective

Policing in the United States presently consists of over 794,000 sworn officers and is projected to rise to 853,000 officers by 2020 [1]. Police officers have high rates of cardiovascular disease (CVD), yet reasons for this finding are not fully understood. Evidence suggests that overweight and obesity may be even more common among police officers than in the general population [2]. As this excess may partly explain the high rates of CVD, we evaluated the association between waist circumference, an indicator of central adiposity, and brachial artery reactivity (BAR), a subclinical marker of CVD. BAR is a measure of the dilation of the brachial artery after occlusion using a blood pressure cuff. Lower BAR indicates dysfunction and early-stage atherosclerosis [3]. Knowledge of associations between obesity and CVD [4–7] leads us to hypothesize that larger waist circumference may be associated with lower BAR.

## 2. Methods

Our study combined data from two cross-sectional studies of police officers in a midsized urban department. The first study, in 1999-2000, included a random sample of 115 officers from 934 employed officers, with oversampling for women. Participation was 100%. The second study, in 2001–2003, included 100 officers, 87 of whom were examined previously. Participants were sworn officers and willing to participate. The studies were approved by The State University of New York at Buffalo Institutional Review Board and the National Institute for Occupational Safety and Health Human Subjects Review Board [8]. Our sample included 70 officers, after excluding 17 officers with missing data for waist circumference or BAR. Data were from 1999-2000, except for BAR which was measured during the second study.

Waist circumference was the mean of two measurements, in centimeters (cm), taken while standing and after exhalation. Body mass index (BMI) and abdominal height were also measured but were not selected because BMI does not distinguish between fat- and lean-mass [9] and because standardized criteria for abdominal obesity (≥102 cm in men, ≥88 cm in women) are more clearly defined for waist circumference [10]. BAR was measured with flow-mediated dilation (FMD), a functional measure of subclinical CVD involving compression of the brachial artery. Arterial diameter was monitored continuously using standardized [11], noninvasive ultrasound scans during the eight-minute procedure: one-minute baseline, four-minute occlusion with a blood pressure cuff, and three-minute period after cuff deflation. BAR was evaluated as percent change in diameter from baseline to maximum diameter following cuff deflation.

Analyses of variance and covariance were used to estimate and describe mean BAR across tertiles of waist circumference. Linear regression assessed the trend, *P* < 0.05. Models were sex stratified and adjusted for age, smoking status (ever/never), and physical activity (composite of hours per week and intensity) which were obtained by self-administered questionnaire. Based on biological plausibility, tests for interaction were conducted for alcohol intake (drinks per week), smoking status, and occupational stress (traumatic police incidents in past year), *P* ≤ 0.20. Due to limited sample size, effect modification was evaluated among men and women combined. Analyses were conducted using SAS, 9.3 (SAS Inc., Cary, NC, USA).

## 3. Results

Mean age for the 70 officers in our study was 43.9 years, and 57.1% were men (Table 1). A greater percentage of women reported current or former smoking. Reported average alcohol intake was less than 3.5 drinks per week. Approximately, 27% of the officers had abdominal obesity (waist circumference ≥102 cm in men and ≥88 cm in women). The mean percent change in BAR was similar for men (4.57%) and women (4.16%). Among several demographic, lifestyle, and CVD risk factors, the only statistically significant difference between study participants (*n* = 70) and nonparticipants (*n* = 35) was in women where participants were older on average.

Adjusted mean percent change in BAR decreased (5.96%, 4.26%, and 3.37%; *P* trend = 0.06) across increasing waist tertiles (80–89.4, 89.5–97.9, and 98–126 cm) among men (Table 2). The trend was not statistically significant in women. Effect modification was then assessed among all officers in models stratified on median alcohol intake (test for interaction: *P* = 0.30). Officers who had intake above the median (0.88 drinks per week) had a significant decline in adjusted mean BAR (5.56%, 5.20%, and 2.12%; *P* = 0.01) across increasing waist tertiles, whereas officers with lower intake had a nonsignificant increase. Tests for statistical interaction involving smoking and traumatic police incidents were not statistically significant.

## 4. Discussion

Although previous studies demonstrate associations between obesity and CVD [4, 5], an association between waist circumference and BAR was observed only in men in our study, even though similar percentages of men and women had an elevated waist circumference. Given that these women were largely of premenopausal age, estrogen could have been an effect modifier [12]. However, the statistical interaction with sex was not statistically significant (*P* = 0.22). The results for alcohol intake were interesting because reported intake was fairly low. Yet, the lowest adjusted mean percent change in BAR was in officers with higher intake and high waist circumference, and, though nonsignificant, the largest change was in officers with lower intake and high waist circumference. Despite assurances of confidentiality, alcohol intake may have been underreported. Although occupational stress, represented as traumatic police incidents, was not an effect modifier in our study, it may be in the causal chain as it may lead to greater central deposition of adipose tissue with increased cortisol levels [13].

Although few studies have focused on this association, our results are similar to those from a large community-based study that also identified an inverse association between waist circumference and BAR, but the association was present in men and women unlike our results which are based on a smaller sample size [6]. In a study of healthy male firefighters, indexes of adiposity were associated with vascular dysfunction, but not specifically with BAR [7]. Neither of these studies specifically examined the effect of alcohol intake on the association between waist circumference and BAR. A larger, more detailed investigation may be beneficial in future studies, particularly with respect to intake patterns including both frequency and intensity. Together, these characteristics have been associated with the distribution of body fat [14].

Strengths of our study include standardized measurement of waist circumference and BAR and oversampling for women. However, lifestyle factors were self-reported and could have changed somewhat over the two- to three-year period. The relatively small sample size and cross-sectional design are potential limitations. Use of data from the two study periods could have resulted in associations that were underestimated, but the average percent change in individual body weight between the studies was rather small at 2.3%.

## 5. Conclusion

Results indicate that in this sample of police officers, larger waist circumference was associated with lower BAR in men, and this association was stronger for officers who consumed more alcohol, a CVD risk factor. The FMD procedure is a research tool that can be useful in characterizing relationships between CVD risk factors, such as central adiposity, and subclinical disease in populations with increased CVD risk [15]. Future studies investigating the relationship between waist circumference and BAR would benefit from a larger sample size and prospective design. Such studies may provide evidence that could be useful in planning intervention studies to determine whether public health efforts to reduce waist circumference may lead to improvements in BAR and thus lower CVD risk.

## Figures and Tables

**Table 1:** Demographic, lifestyle, occupational, and physical characteristics of participating police officers.

Characteristic	Men	Women	All	*P* value^a^
	(*n* = 40)	(*n* = 30)	(*n* = 70)	
Age, mean (SD), y	43.88 (9.16)	43.90 (5.99)	43.89 (7.91)	0.99
Race/ethnicity				
White	34 (85.0)	22 (73.3)	56 (80.0)	
Black	3 (7.5)	8 (26.7)	11 (15.7)	0.04
Hispanic	3 (7.5)	0 (0.0)	3 (4.3)	
Education				
≤High school/GED	6 (15.0)	6 (20.0)	12 (17.1)	
College, <4 y	11 (27.5)	11 (36.7)	22 (31.4)	0.50
College, ≥4 y	23 (57.5)	13 (43.3)	36 (51.4)	
Smoking				
Ever	17 (42.5)	19 (63.3)	36 (51.4)	0.08
Never	23 (57.5)	11 (36.7)	34 (48.6)	
Physical activity^b^, mean (SD), h/wk	7.11 (10.77)	6.76 (9.01)	6.96 (9.98)	0.88
Physical activity index, (h/wk) × intensity^c^				
0–1.50	9 (22.5)	9 (30.0)	18 (25.7)	
1.51–6.50	13 (32.5)	4 (13.3)	17 (24.3)	0.12
6.51–14.50	7 (17.5)	11 (36.7)	18 (25.7)	
≥14.51	11 (27.5)	6 (20.0)	17 (24.3)	
Alcohol intake, drinks/wk				
All participants, mean (SD)	3.45 (4.68)	2.04 (3.31)	2.84 (4.18)	0.16
0	7 (17.5)	11 (36.7)	18 (25.7)	
<1	11 (27.5)	8 (26.7)	19 (27.1)	0.23
1–5	15 (37.5)	9 (30.0)	24 (34.3)	
≥6	7 (17.5)	2 (6.7)	9 (12.9)	
Traumatic police incidents, mean (SD), events/y	4.68 (2.19)	3.30 (2.15)	4.09 (2.26)	0.01
Waist circumference, cm				
All participants, mean (SD)	95.06 (10.04)	81.95 (10.77)	89.44 (12.18)	<0.001
Normal (<102 in men, <88 in women)	29 (72.5)	22 (73.3)	51 (72.9)	0.94
Abdominal obesity (≥102 in men, ≥88 in women)	11 (27.5)	8 (26.7)	19 (27.1)	
Brachial artery reactivity, mean (SD), % increase	4.57 (3.47)	4.16 (3.53)	4.39 (3.48)	0.63

SD: standard deviation; cm: centimeters.

^a^Test compares gender-specific values. *P* values from chi-squared test or Fisher's exact test for categorical variables and from *t*-test for continuous variables.

^b^Moderate intensity or greater.

^c^Intensity categorized as moderate, hard, and very hard.

**Table 2:** Mean percent change in brachial artery reactivity (BAR) by tertiles of waist circumference in police officers.

Waist tertile^a^	*N *	Unadjusted		Multivariate adjusted^b^	
		Mean (SD)	*P* value^c^	Mean (SE)	*P* value^c^
Men					
Low	14	5.61 (4.47)		5.96 (0.94)	
Medium	13	4.69 (2.97)	0.04	4.26 (0.95)	0.06
High	13	3.32 (2.42)		3.37 (0.94)	
Women					
Low	10	4.99 (2.95)		4.99 (1.16)	
Medium	10	3.32 (4.78)	0.83	3.47 (1.16)	0.87
High	10	4.18 (2.67)		4.02 (1.16)	
Waist tertile by alcohol intake ≤ median^d^					
Low	12	3.55 (3.23)		3.31 (1.16)	
Medium	13	4.97 (5.46)	0.74	4.40 (1.14)	0.27
High	12	5.11 (2.57)		5.96 (1.19)	
Waist tertile by alcohol intake > median					
Low	12	5.57 (2.75)		5.56 (0.78)	
Medium	9	4.82 (3.32)	0.01	5.20 (0.95)	0.01
High	12	2.40 (1.73)		2.12 (0.81)	

SD: standard deviation; SE: standard error.

^a^Waist tertiles were 80–89.4, 89.5–97.9, and 98–126 cm for men, 64.5–74.9, 75–86.9, and 87–110.1 cm for women, and 64.5–85.1, 85.2–92.4, and 92.5–126 cm for all police officers.

^b^Adjusted for age, smoking, and physical activity.

^c^ *P* values are from linear regression models.

^d^Median value was 0.88 drinks per week. *P* value for interaction with alcohol was 0.30.
